# Macromolecular Architecture in the Synthesis of Micro- and Mesoporous Polymers

**DOI:** 10.3390/polym16233267

**Published:** 2024-11-24

**Authors:** Ilsiya M. Davletbaeva, Oleg O. Sazonov

**Affiliations:** Technology of Synthetic Rubber Department, Kazan National Research Technological University, 68 Karl Marx Str., 420015 Kazan, Russia; sazonov.oleg2010@gmail.com

**Keywords:** macromolecular architecture, synthesis, microporous polymers, mesoporous polymers

## Abstract

Polymers with micro- and mesoporous structure are promising as materials for gas storage and separation, encapsulating agents for controlled drug release, carriers for catalysts and sensors, precursors of nanostructured carbon materials, carriers for biomolecular immobilization and cellular scaffolds, as materials with a low dielectric constant, filtering/separating membranes, proton exchange membranes, templates for replicating structures, and as electrode materials for energy storage. Sol–gel technologies, track etching, and template synthesis are used for their production, including in micelles of surfactants and microemulsions and sublimation drying. The listed methods make it possible to obtain pores with variable shapes and sizes of 5–50 nm and achieve a narrow pore size distribution. However, all these methods are technologically multi-stage and require the use of consumables. This paper presents a review of the use of macromolecular architecture in the synthesis of micro- and mesoporous polymers with extremely high surface area and hierarchical porous polymers. The synthesis of porous polymer frameworks with individual functional capabilities, the required chemical structure, and pore surface sizes is based on the unique possibilities of developing the architecture of the polymer matrix.

## 1. Introduction

Synthesis of organic polymers with micro- and mesoporous structure is relevant in polymer chemistry. According to IUPAC recommendations, microporous polymers are those with a pore diameter of less than 2 nm; for mesoporous polymers, the pore size is in the range of 2–50 nm; macroporous polymers are characterized by a pore size of more than 50 nm [1,2,3,4,5]. In the most cases, micro- and mesoporous polymers have permanent interconnected voids and achieve practical significance due to the permeability of the pores for liquids or gases. The most important structural characteristics of porous polymers include geometry, size, functionality of the pore surface, and the structure of the polymer framework, including composition, topology, and functionality [6,7]. Pores can have spherical, tubular, and network (disordered or ordered) morphology. To evaluate the pore structure, such parameters as surface area, functionality of the polymer framework, and pore surface are used [8,9].

The advantage of the organic polymer framework is their relatively low density. The main areas of application of porous polymers include the following: materials for gas storage and separation, encapsulating agents for controlled drug release, as carriers for catalysts and sensors, templates for creating nanostructured carbon materials, carriers for biomolecular immobilization and cell scaffolds, materials with a low dielectric constant, filtration/separation membranes, proton exchange membranes, templates for replicating structures, electrode materials for energy storage, among many other applications.

**Figure 1:**
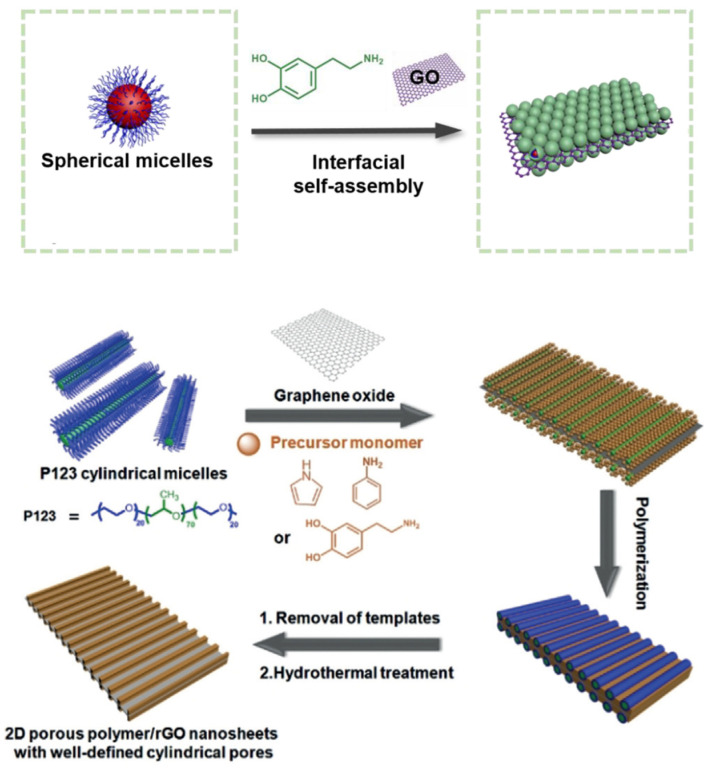
Schematic diagram of 2D interfacial self-assembly toward mPDA/rGO nanosheets with tunable pore structures (reprinted with permission from [10], 2019, American Chemical Society). Illustration of the fabrication of 2D mesoporous polymer/rGO nanosheets with cylindrical pores by the interface self-assembly strategy. Note that the cylindrical micelles and pores should be freely curved in practice; in the diagram they are drawn as straight structures for convenience (reprinted with permission from [11], 2019, Wiley).

Sol–gel technologies, track etching, and template synthesis are used to obtain polymers, including in micelles of surfactants and microemulsions [10,11] (Figure 1) and sublimation drying. The listed methods allow obtaining pores with variable shapes and sizes of 5–50 nm and achieving their narrow size distribution.

However, all these methods are technologically multi-stage and require the use of consumable components, which leads to low efficiency of using the starting materials, and the final product does not have micropores. These drawbacks are absent in the methods of direct synthesis of porous polymers, which make it possible to obtain micro- and mesoporous polymers with an extremely high surface area and hierarchical porous polymers.

The processes used to form pores can essentially be reduced to two general categories [12]: some are based on networks of highly cross-linked polymer chains or are due to inefficient packing of very rigid and curved non-cross-linked polymer chains; while others are the result of phase separation. In the first category, it becomes possible to form microporous polymers; in the second category, to form meso- or macroporous polymers. It is possible to create hierarchical porous polymers with meso- and macropores by combining micropores formed as a result of inefficient packing of a network of rigid polymer chains. With a decrease in the rigidity of the polymer chains of the framework, micropores can be destroyed. To ensure the stability of the structure of microporous polymers, the monomers used for their synthesis in one of the variants must have multifunctional reaction centers, rigid or curved structures capable of forming a network or a linear polymer framework [13].

## 2. Porous Organic Polymers

Porous organic polymers (POPs) are organic macromolecules, the characteristic features of which include tunable porosity, low density, high chemical and thermal stability, variable composition, high specific surface area, and the possibility of post-functionalization [14,15,16,17,18,19,20].

The main types of POPs, depending on the nature of the initial components and methods of their synthesis, include porous aromatic frameworks (PAFs), including covalent organic frameworks (COFs), covalent triazine frameworks (CTFs), hypercrosslinked polymers (HCPs), including hypercrosslinked polystyrene (HCPSt), and conjugated microporous polymers (CMPs).

Due to the variety of methods for synthesizing POPs, unique opportunities arise for developing the architecture of the polymer matrix and creating numerous porous polymers with individual functional capabilities of the framework and pore surface.

### 2.1. Aromatic Porous Structures

Porous aromatic frameworks (PAFs) are constructed entirely from organic building blocks linked via stable carbon–carbon covalent bonds [21]. Due to their structural features, PAFs allow for large surface areas and the ability to significantly influence their structure. PAFs exhibit high chemical and thermal stability and, due to their numerous accessible internal surfaces, stable framework, and subnanometer pores, are used as functional materials for adsorption, separation, catalysis, and as solid electrolytes [22,23,24,25,26,27,28,29,30,31,32,33,34]. Due to their robust porous structure, the existence of interconnected channels, and the ability for electrolyte ions to migrate through the channels, PAFs have proven to be very effective for use in electrochemical processes [35,36], including as electrode materials [37,38,39,40,41].

**Figure 2:**
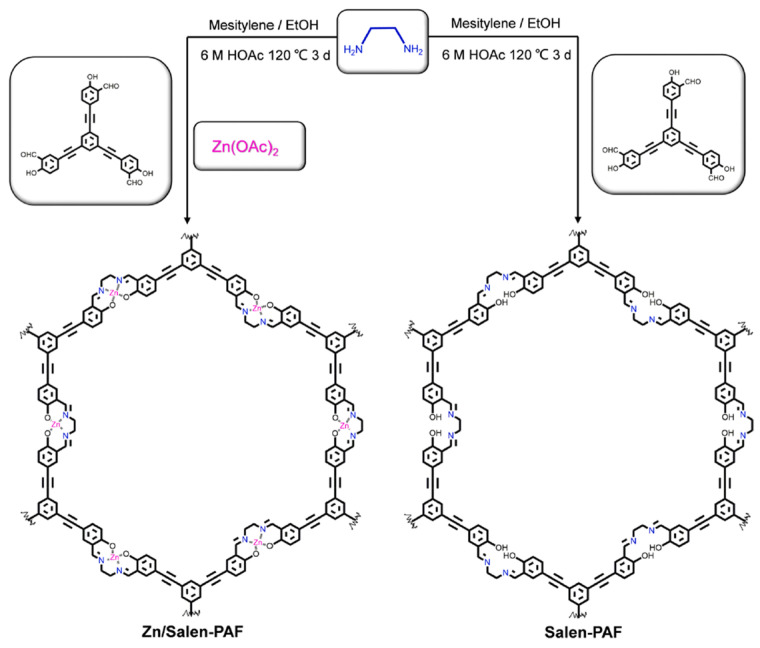
Synthesis scheme for the Zn/Salen-PAF and Salen-PAF. (Reprinted with permission from [42], Copyright 2023, Elsevier).

PAFs are constructed through a metal directed method designed and synthesized by the Zn/Salen-based porous aromatic frame-work (Zn/Salen-PAF) (Figure 2) [42]. Zn/Salen-PAF was investigated as an anode material for use in lithium–ion batteries. The electrochemical performance of Zn/Salen-based PAFs is significantly affected by the coordination binding of zinc ions. Thus, Zn/Salen-PAFs exhibit high charge–discharge capacity, cyclic stability, and speed characteristics.

Salen consists of double fragments of Schiff bases and belongs to the type of ligands with a tetracoordinate structure. As a result, the Salen ligand exhibits the ability to coordinate binding with transition metal ions [43,44,45]. Due to the peculiarities of their structural characteristics [46,47], salen complexes are promising as reusable photocatalysts and other areas of application.

**Figure 3:**
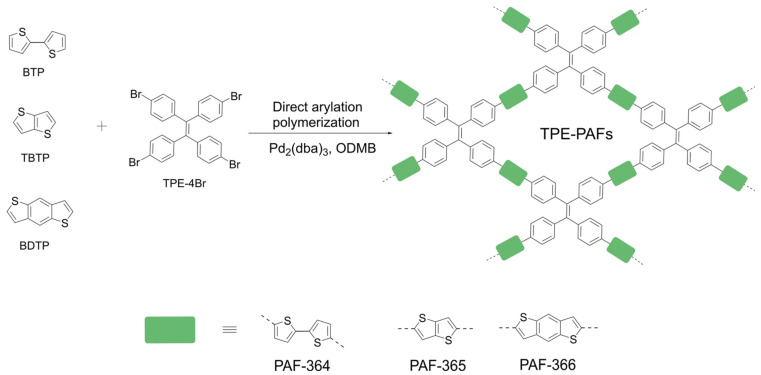
Synthesis of PAF-364, PAF-365, and PAF-366. (Reprinted with permission from [48], 2024, The Royal Society of Chemistry).

PAFs can provide a green and sustainable alternative for conventional metal-based photocatalysts as they are visible light-active and reusable. A novel photoactive tetraphenylethylene-based porous aromatic framework photocatalyst linked with thiophene units in an alternating donor–acceptor fashion (TPE-PAFs) was synthesized [48] (Figure 3). According to photoelectrochemical studies, the existence of thiophene units in the structure of TPE-PAFs make it possible to influence the optical band gap and energy level and thus effectively regulate their photocatalytic activity. The resulting TPE-PAF achieves 99% yields and up to 10 cycles recyclability during photosynthesis of benzimidazoles.

**Figure 4:**
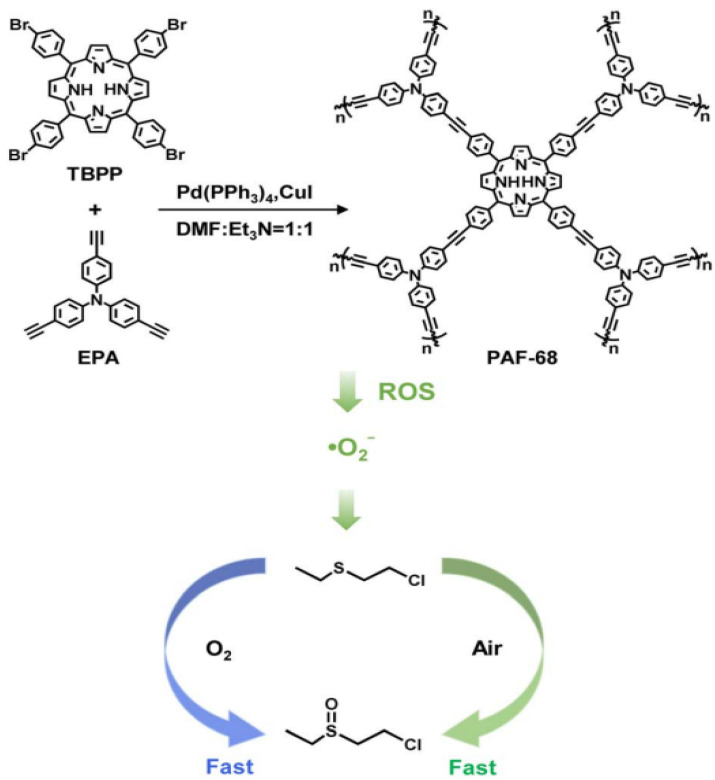
Schemes of the PAF-68 synthesis and photocatalytic detoxification of mustard gas. (Reprinted with permission from [49], 2024, The Royal Society of Chemistry).

A photoactive PAF (PAF-68) with high stability and specific surface area was prepared using *tris*(4-ethynylphenyl)amine (EPA) and *meso*-tetra(p-bromophenyl)porphyrin (TBPP) (Figure 4) [49]. The synthesized PAF-68 was investigated as an efficient agent for photocatalytic aerobic oxidation of a mustard gas simulant. The existence of porphyrin in the structure of PAF-68 results in low exciton binding energy and long decay lifetime when excited by light, both of which would facilitate the generation of ∙O_2_^−^. The mechanism study revealed that the high-energy carbon–carbon bonds in the amorphous aromatic framework of PAF-68 were responsible for the inhibition of photocatalytic degradation of the skeleton and the stability of photocatalytic degradation over several cycles.

**Figure 5:**
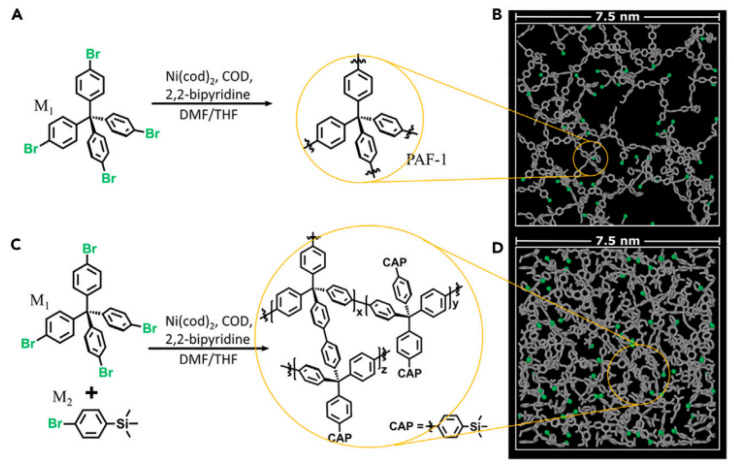
Synthesis scheme of PAF-1 (**A**) Molecular model of PAF-1 with a cross section of 2 nm. Carbon shown in gray, unreacted bromine ends are shown in green (**B**). Synthesis of PAF-1 derivatives with disrupted pore structure via the Ni-catalyzed Yamamoto-type Ullmann coupling of M1 and M2 (**C**). Molecular model of PAF-1 with a cross section of 2 nm. PAF-1 was obtained at a ratio of M2/M1 = 1.8 (**D**). (Reprinted with permission from [50], Copyright 2023, Elsevier).

A strategy of defect engineering capable of modulating the polymer connectivity and porosity of aromatic frameworks was used in the study [50]. Based on the monofunctionalized end-capping monomer (M1), the synthesis of PAF-1 (Figure 5A), which disrupts the topology of the framework, was carried out. The interaction was carried out by coupling via the Ni-catalyzed Yamamoto-type Ullmann (Figure 5). As a result of the formation of less densely cross-linked networks and subsequent pore collapse, more effective adsorption sites for removing 1,4-dioxane from water formed. By increasing the content of M1 in the reaction system, PAFs with a narrower pore size distribution were obtained and the absorption of 1,4-dioxane, an important environmental pollutant, was improved. The molecular modeling confirmed the experimental characteristics and the efficiency of using PAF-1 in selective adsorption separation.

**Figure 6:**
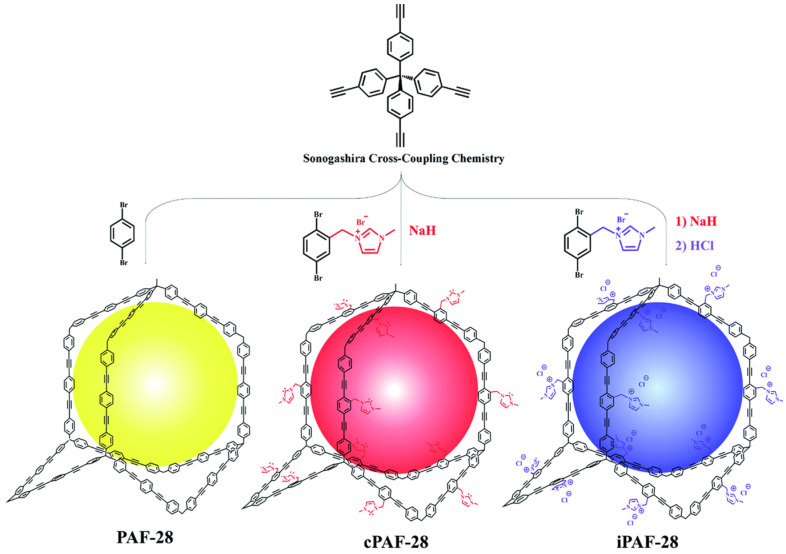
Schematic representation of the synthesis of PAF-28, cPAF-28, and iPAF-28. (Reprinted with permission from [51], 2022, The Royal Society of Chemistry).

Highly porous and thermally stable unfunctionalized PAF-28, imidazolium-functionalized iPAF-28 and carbene-functionalized cPAF-28, which are 3D porous aromatic frameworks, were prepared using Sonogashira coupling reactions (Figure 6) [51]. The synthesized cPAF-28 and iPAF-28 exhibited high selectivity for C_2_H_2_ adsorption from the gas mixture C_2_H_2_/C_2_H_4_, which is 12.2 for cPAF-28 and 15.4 for iPAF-28. The capacity of the C_2_H_2_ is 48 cm^3^ g^−1^ for cPAF-28 and 57 cm^3^ g^−1^ for iPAF-28. For unfunctionalized PAF-28, the selectivity of the C_2_H_2_ adsorption is 1.8, and the capacity of the C_2_H_2_ is 37 cm^3^ g^−1^. A distinctive feature of cPAF-28 and iPAF-28 is also remarkable recyclability for the separation of the C_2_H_2_/C_2_H_4_ gas mixture.

### 2.2. Covalent Organic Frameworks

Covalent organic frameworks (COFs) are a class of crystalline porous organic polymers with highly ordered structures [8]. An important feature of COFs is their structural predesignability, the ability to control functionality and synthesis, including geometric guidance for the structural tiling of extended porous polygons. With a diversity of topologies and linkages, the availability of organic units represents the prospect of developing COFs as a new field of porous organic polymers [8,9,16,17,18,19,20]. The synthesis of COFs as predesigned primary and highly ordered structures is based on polycondensation reactions.

**Figure 7:**
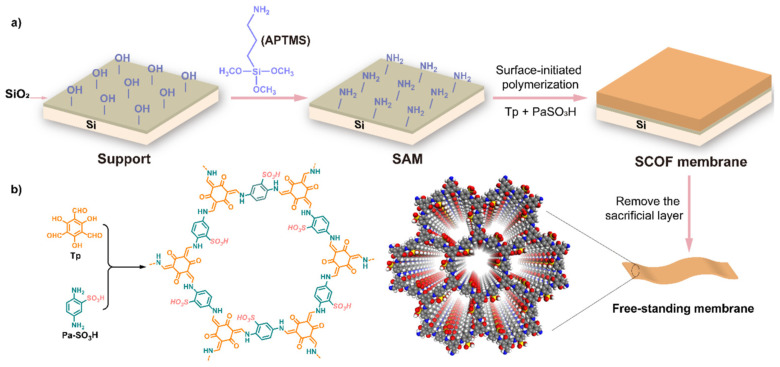
Synthetic scheme (**a**) for the preparation of the SCOF membrane grafted on silicon wafers and the free-standing SCOF membrane; (**b**) Molecular structures for Tp, Pa-SO_3_H and TpPa-SO_3_H. (Reprinted with permission from [52], 2021, Wiley).

The synthesis of COFs with a high content of sulfuric acid groups was described in one study [52]. The proton conductivity of membranes based on them reaches 0.54 S cm^−1^ at 80 °C in pure water. Such crystalline porous organic materials are distinguished by the regularity of channels and individual functionality. Since such structures are insoluble and unprocessable, the surface-initiated polycondensation of trialdehyde and phenylenediamine was used to synthesize sulfone coatings. A feature of this approach is the ability to accurately change the coating thickness from ten to one hundred nanometers by controlling the polymerization time (Figure 7).

### 2.3. Conjugated Microporous Polymers

Due to their unique structure combining π-conjugation with a permanent microporous skeleton, conjugated microporous polymers (CMPs) create the possibility of precise design of the pore environment and the creation of functional porous materials at the molecular level [53,54,55,56,57,58,59,60,61,62,63,64,65,66].

**Figure 8:**
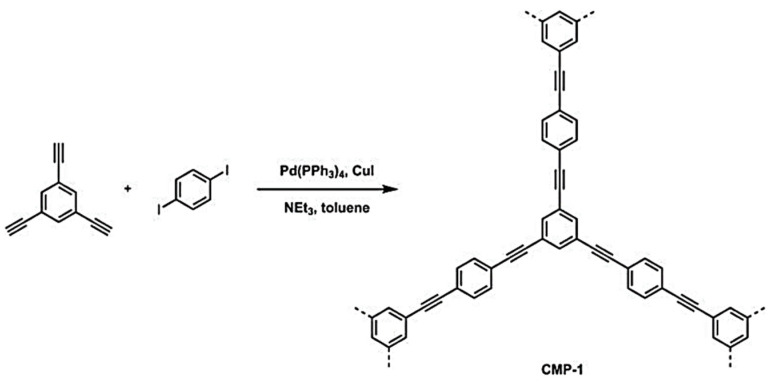
Synthesis of poly(aryleneethynylene) network, CMP-1. (Reprinted with permission from [67], 2007, Wiley).

CMP networks are synthesized using reactions based on two or more monomers with different chemical structures; there are also examples of homocoupling of a single monomer. CMP networks can be formed through such reactive coupling groups as halogens, alkynes, alkenes, nitriles, boronic acids, amine, and aldehydes. CMPs can be prepared using Sonogashira–Hagihara cross-couplings [67]. This reaction couples an aryl halide with an alkyne-containing monomer using a palladium catalyst and a copper cocatalyst (Figure 8).

Other processes used for the synthesis of CMPs include the following: the Suzuki–Miyaura cross-coupling reaction [68], Yamamoto coupling [69], Heck reaction, also known as the Mizoroki–Heck reaction [70,71,72], cyclotrimerization reactions [73], via phenazine ring fusions [74,75], Schiff base condensations [76,77], heterocycle linkages [78,79], alkyne metathesis [80], oxidative coupling [81,82], and Buchwald–Hartwig amination [83,84]. The microporous structure of CMPs and the presence of conjugated backbones provide excellent physicochemical properties, create a large pore space to accommodate carriers and can inhibit the dissolution of the materials.

Due to the use of a wide range of synthetic building blocks and the ability to chemically modify CMPs and tailor their functionality to ever-changing environments and needs, a variety of CMPs have been developed. This has allowed the development of CMPs for applications such as gas storage and separations [85,86,87,88,89,90], heterogeneous catalysis [91,92,93,94,95,96], light emittance [97,98,99,100,101,102], photovoltaic materials [103,104,105], chemosensing [106,107,108,109,110,111], and biological [112,113,114,115,116,117,118,119].

**Figure 9:**
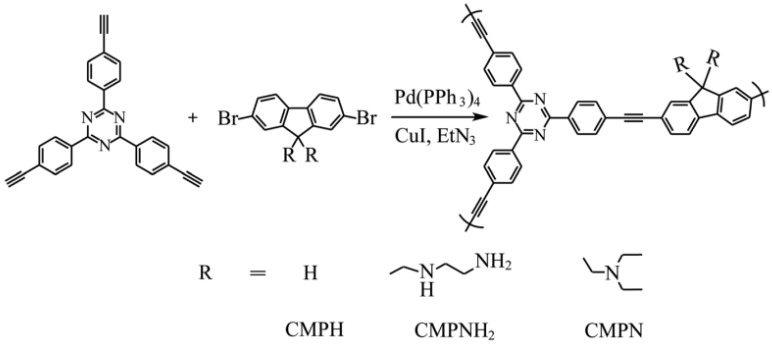
Synthetic route of CMPs. (Reprinted with permission from [126], 2020, The Royal Society of Chemistry).

A promising area of the application for CMPs is the encapsulation of chemicals [120,121,122,123,124,125]. Amine-functionalized fluorescent conjugated mesoporous polymers (CMPs) were prepared using the Sonogashira–Hagihara coupling reaction (Figure 9) [126]. CMP studies are aimed at real-time detection of iodine by its efficient capture. In the synthesis of CMPs, 2,7-dibromofluorenes with ethylenediamine or diethylamine were used as linker monomers to create flexible binding sites. The compound 2,4,6-tris(4-ethynylphenyl)-1,3,5-triazine was chosen to create a rigid skeleton. CMPs prepared from ethylenediamine designated as CMPNH_2_, and CMPs prepared using diethylamine are called CMPN.

**Figure 10:**
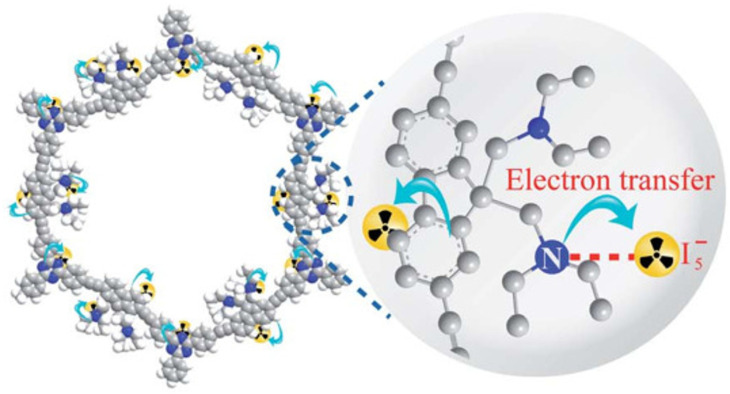
Schematic illustration of the iodine uptake mechanism of CMPN. (Reprinted with permission from [126], 2020, The Royal Society of Chemistry).

CMPN prepared using N,N-diethylpropylamine allows achieving the highest iodine absorption values among all CMP-based materials known to date (Figure 10) [126]. CMPN is able to efficiently capture iodine from both iodine vapor and iodine solutions. Highly sensitive iodine detection on test paper with a CMPN-based selective layer was observed even at 4 °C under low vapor pressure conditions (16.8 Pa). The fluorescence quenching amplification of the conjugation skeleton, CMPN is also the reason for excellent stability against β- or γ-ray irradiation

A promising direction is the use of CMP in photocatalytic hydrogen evolution, since inorganic semiconductors exhibit low activity in visible light and, due to their chemical structure, there are issues of tunability [127,128,129]. The ability to control the microstructure and electronic properties of CMP creates serious advantages for these materials for photocatalytic H_2_ evolution [130,131,132,133,134,135,136,137,138].

**Figure 11:**
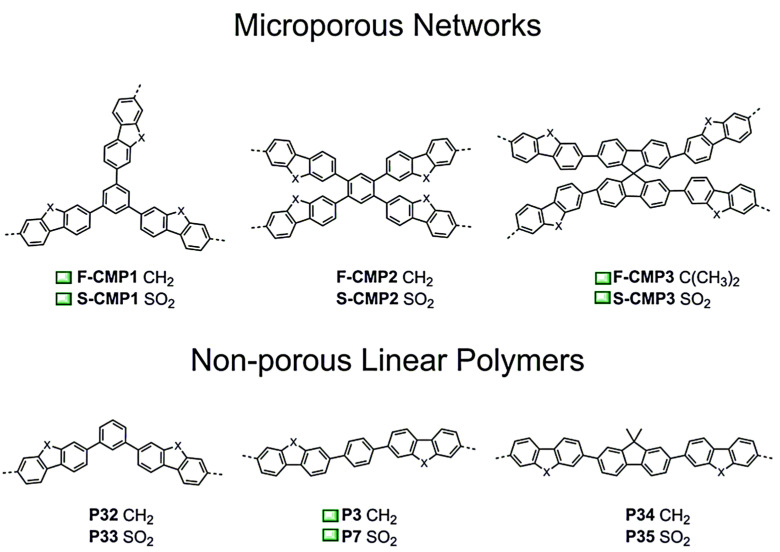
Conjugated microporous polymer networks (**1st line**), linear CMP equivalent (**2nd line**). The green square indicates the most productive (under visible light conditions) photocatalysts. (Reprinted with permission from [138], 2021, American Chemical Society).

However, it has not been established whether porosity is one of the main reasons for photocatalytic H_2_ evolution. Thus, in [138] three series of CMPs were investigated in comparison with their linear structural analogues (Figure 11). The optimal porous or nonporous morphology depends on the linkers used in the polymer synthesis. It was found that both porous CMPs and nonporous linear analogs generally exhibit similar efficiency in photocatalytic H_2_ evolution. Moreover, optical gaps and light absorption profiles observed for porous and non-porous polymers turned out to be very close. Factors such as swelling degree, Pd residues, wettability, and particle size also affect the photocatalytic activity of these polymers. The advantages of porous morphology increase access to catalytically active sites of the CMP, which is further benefited during swelling. Conversely, the reduction in charge-mobility may be due to the presence of highly twisted structures in the CMP linkers and any mass transfer benefits that arise from porosity may be negated.

When designing the synthesis of CMPs for photocatalytic H_2_ evolution, one important parameter to consider is ensuring efficient conjugation in the CMP. Planarization of the structure is reduced by the existence of twisted aromatic units, and a decrease in the conjugation throughout the polymer often leads to a decrease in the photocatalytic activity. In turn, compared to nonporous linear polymers, porous networks can lead to an increase in the twisting of aromatic units. The porous morphology increases the penetration of water and its contact with the catalytically active sites, increasing the mass transfer efficiency.

There are many reports of the use of CMPs as excellent materials for energy storage [139,140,141,142,143,144,145,146,147,148,149,150,151,152].

**Figure 12:**
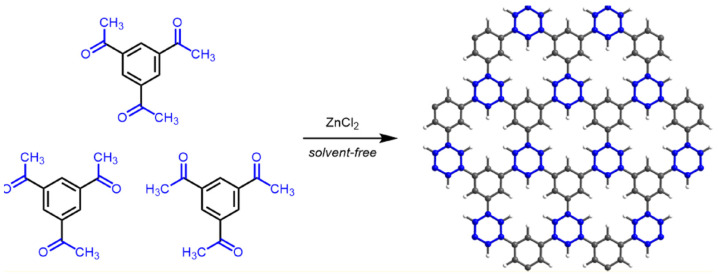
Ionothermal trimerization of methyl ketones to prepare conjugated microporous polymers. (Reprinted with permission from [153], 2021, American Chemical Society).

A new solvent-free synthesis strategy of CMP and PAF involving ionothermal cyclotrimerization of methyl ketones via the aldol reaction was reported [153]. To obtain highly conjugated microporous materials in molten zinc chloride, polymerization of 1,3,5-triacetylbenzene and tetrakis(4-acetylphenyl)methane was carried out (Figure 12). The obtained polymers were investigated as promising materials for electrical energy storage applications. For CMP obtained from 1,3,5-triacetylbenzene charge storage capacities reach 172 F g^−1^, exceeding similar values for the known commercial supercapacitor carbon.

**Figure 13:**
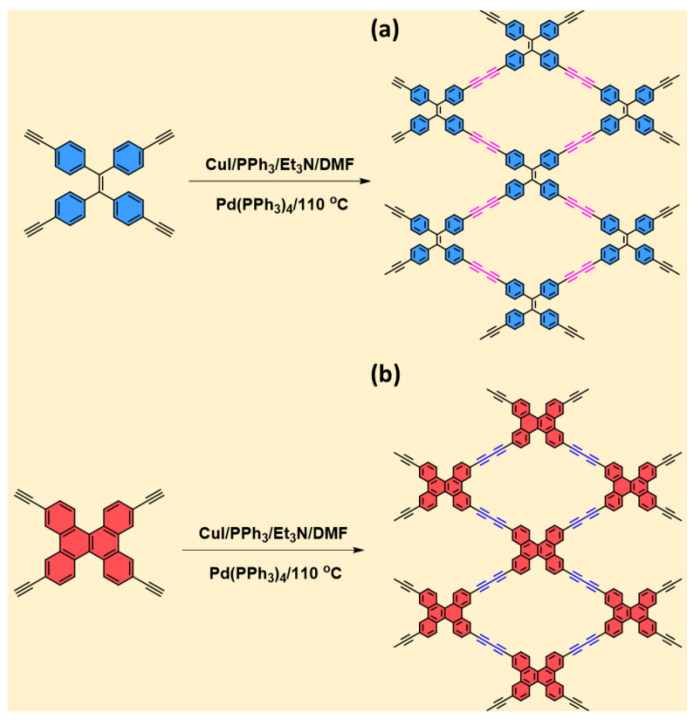
Schematic method for the synthesis of the (**a**) TPE-Diyne CMP and (**b**) TBN-Diyne CMP. (Reprinted with permission from [154], 2024, The Royal Society of Chemistry).

In [154], two robust diyne-linked CMPs derived from TPE-Diyne and TBN-Diyne were successfully synthesized using the Pd-catalyzed coupling technique for their respective monomers (TPE-TB and TBN-TB) (Figure 13). Based on TGA data, TPE-Diyne and TBN-Diyne CMPs exhibited Td10 values of up to 380 and 360 °C, respectively, under a nitrogen atmosphere. Moreover, it was noted that the CMPs exhibited distinct microporosity and significant specific surface areas, with the TPE-Diyne CMP measuring 428 m^2^ g^−1^ and the TBN-Diyne CMP reaching 256 m^2^ g^−1^. With a specific capacitance of 39 F g^−1^ and an impressive capacitance retention of 98% after 2000 cycles [measured at 10 A g^−1^], the TPE-Diyne CMP demonstrates significant promise for supercapacitor applications.

CMPs exhibiting semiconductor properties have been widely employed as heterogeneous photocatalysts. Photogenerated charge carrier separation and transfer are crucial factors influencing the performance of such photocatalysts [155,156,157,158,159,160,161,162,163,164].

**Figure 14:**
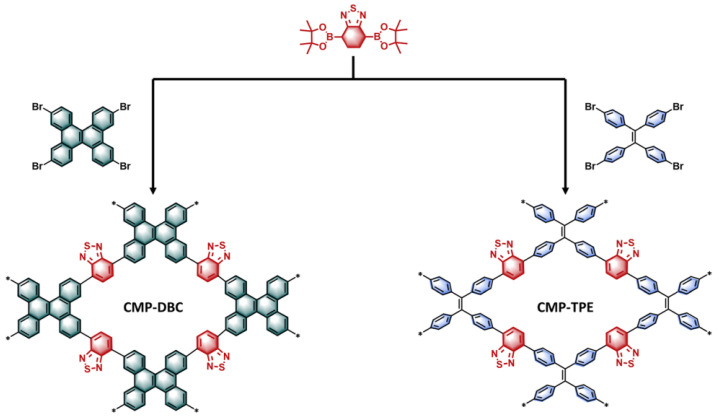
The synthetic routes for the two polymers and the notional structures. (Reprinted with permission from [165], 2024, The Royal Society of Chemistry).

The CMP dibenzo[g,p]chrysene (CMP-DBC), which has a planar structure, and the CMP tetraphenylethylene (CMP-TPE), which is characterized by a non-planar structure (Figure 14) have been developed [165]. CMP-DBC and CMP-TPE were used to study the effect of planarity on the photocatalytic properties of CMP-based materials. The reason for the studies was the fact that single bonding of electron-acceptor units by single carbon–carbon bonds can lead to the occurrence of steric hindrances and the formation of large dihedral angles. As a result, the transport of charge carriers hindered, and the photocatalytic properties of CMP are limited due to the recombination of photogenerated electron-hole pairs. The theoretical modeling of CMP-DBC and CMP-TPE was carried out, and it was found that, in the case of a planar structure, a significantly smaller dihedral angle for the electron donor occurs, amounting to 18.41° compared to CMP-TPE, where the dihedral angle reaches 47.81°, and the exciton binding energy for CMP-DBC is 108 meV and is also lower compared to CMP-TPE, for which it reaches 126 meV. The exciton binding energy was obtained using temperature-dependent fluorescence spectroscopy. Due to the relatively small donor–acceptor dihedral angles in the CMP-DBC structure, the migration of charge carriers becomes easier. As a result, the high-speed radiative recombination was effectively suppressed, and superior photocatalytic performance was observed. Thus, a 60% yield in the photocatalytic dehalogenation reaction was obtained for CMP-TPE; while for CMP-DBC, close to 100% halide product yield was recorded.

**Figure 15:**
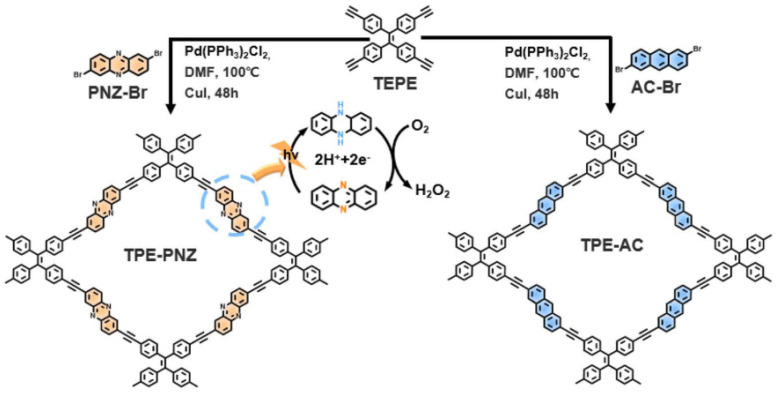
Schematic illustration of the synthetic routes towards TPE-PNZ and TPE-AC from their corresponding precursors. (Reprinted with permission from [166], 2024, The Royal Society of Chemistry).

Solar fuel generation largely depends on the sustainability of the solar-driven hydrogen peroxide production process. With their vast structural versatility and extended π-conjugation, CMPs are promising photocatalysts for solar-driven hydrogen peroxide generation from water and oxygen, though enhancing their efficiency is challenging. A new CMP-based photocatalyst for H_2_O_2_ production was obtained by integrating a phenazine fragment into the CMP (TPE-PNZ) structure [166] (Figure 15). The main factor enhancing the photocatalytic activity of CMP (TPE-PNZ) in hydrogen peroxide synthesis was based on the efficiency of oxidation-reduction cycles and electron transfer processes occurring during reversible redox dynamics between phenazine and dihydrophenazine. Studies have shown that this reversible process enhances oxygen adsorption and its subsequent reduction, and significantly leads to a decrease in the energy barrier in hydrogen peroxide formation. The electrons are photogenerated by interconversion stored by phenazine. Subsequent conversion of phenazine to dihydrophenazine results in the reduction of O_2_ to H_2_O_2_. Then phenazine formed again, significantly facilitating charge transfer and softening charge recombination. The productivity of using TPE-PNZ in obtaining hydrogen peroxide is 5142 mmol/g∙h, and the efficiency of converting solar energy into chemical energy reaches 0.58%. At the same time, there is no need to use sacrificial agents.

**Figure 16:**
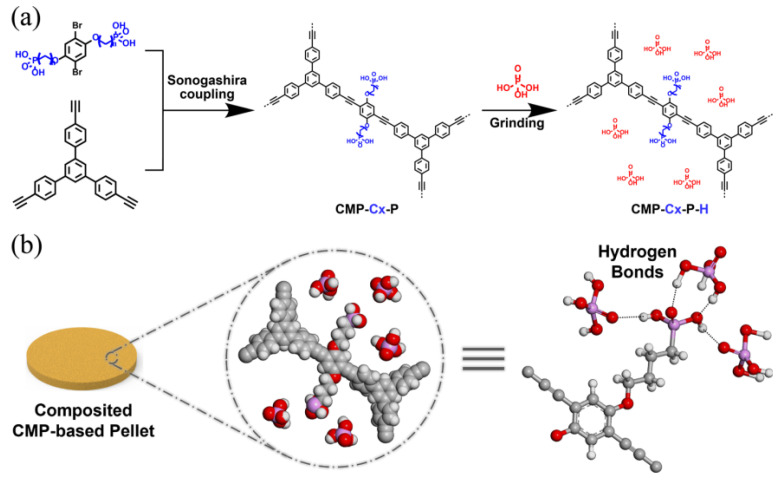
(**a**) Illustration of the synthesis of the phosphonated CMPs composited with H_3_PO_4_. (**b**) Demonstrating the assembly of hydrogen-bonding networks. Pink, P; red, O; white, H; grey, C. (Reprinted with permission from [167], 2023, The Royal Society of Chemistry).

Proton exchange membranes are one of the main components of fuel cells, which are capable of efficiently converting chemical fuel into electrical energy. The development of new proton-conducting polyelectrolytes has become increasingly important in the last decade due to high proton conductivity, it is necessary to achieve multi-environmental conditions in membranes. High proton conductivity in multi-environmental conditions is exhibited by alkoxy phosphonic acid-functionalized CMPs, the synthesis of which was carried out using Sonogashira–Hagihara cross-coupling and side-chain engineering (Figure 16) [167]. The anhydrous proton conductivity of phosphonated CMPs composited with *ortho*-phosphoric acid at −40 °C was 1.15 × 10^−5^ S cm^−1^ and at 130 °C it reached 2.15 × 10^−2^ S cm^−1^. The proton conductivity at 30 °C and under 32% relative humidity was 1.87 × 10^−2^ S cm^−1^. With an increase in temperature to 90 °C and relative humidity to 98%, the conductivity reaches 1.87 × 10^−2^ S cm^−1^. It was shown that the achievement of high proton conductivity of the samples of the phosphonated CMPs was due to their hydrophilic nature, high stability and mobility of the side chain, and the many molecules of *ortho*-phosphoric acid involved in their structural organization.

### 2.4. Microporous Polymers Based on Covalent Triazine Frameworks

Covalent triazine framework CTFs have a 1,3,5-triazine ring covalently bonded to organic units with remarkable photophysical properties [168,169,170,171]. CTFs are promising for using in various photocatalytic reactions [172,173,174] and are potentially useful in such applications as gas separation and storage [175], energy storage [176], photocatalysis [177], and heterogeneous catalysis [178]. The problem of using covalent triazine frameworks is largely due to the multitude of defects in their amorphous state. The influence of defects on the structure of the electron zone is due to the recombination of photogenerated electron and hole pairs in abundantly available trap sites [179]. The best characteristics in optoelectronics are achievable for highly crystalline materials since their structure contains much fewer trap sites for photoinduced charges [180].

Although the structures of CTFs and CMPs differ in many ways, the existence of extended π-conjugation in both cases leads to considered covalent triazine frameworks as the CMPs subclass.

Indeed, the excellent surface area of highly crystalline microporous semiconductor materials has great potential for use; however, obtaining CTFs with ultrastrong covalent bonds of carbon atoms of aromatic moieties with nitrogen atoms in the triazine structures is a major challenge [181,182,183,184,185,186].

**Figure 17:**
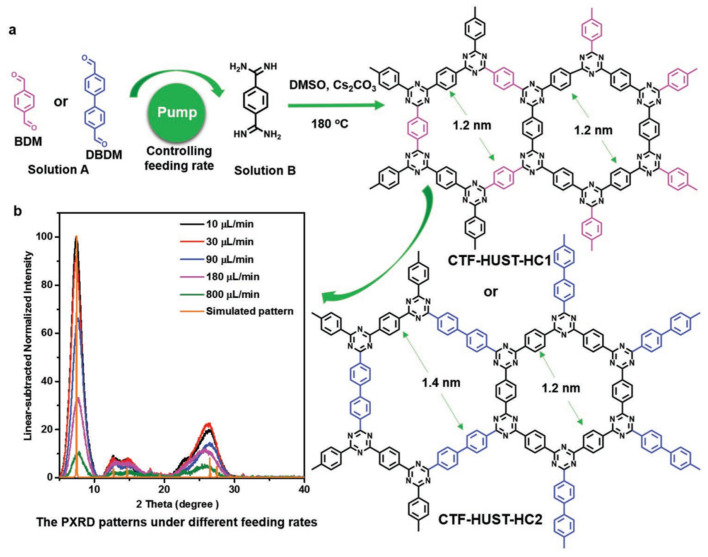
The general strategy: (**a**) the nucleation rate and crystal growth were controlled by lower feeding rate to synthesize highly crystalline CTF-HUST-HC1 and CTF-HUST-HC2. “HUST” is the abbreviation of “Huazhong University of Science and Technology”. “HC” is the abbreviation of “High Crystal”. (**b**) PXRD patterns of CTF-HUST-HC1 samples under different feeding rates, normalized at 2θ = 38° for clarity. (Reprinted with permission from [180], 2019, Wiley).

The crystallization process in the synthesis of covalent triazine frameworks can be readily governed by directly controlling the monomer feeding rate. The chosen strategy resulted in the production of highly crystalline CTF-HUST-HC1 (Figure 17) [180]. Since CTF-HUST-HC1 is characterized by abundant exposed {001} crystal facets, this sample was used to study photovoltaic properties and the effect of high crystallinity. Indeed, the highly ordered structure of CTF-HUST-HC1 was found to be responsible for a significantly higher performance in photocatalytic nitric oxide removal compared to less crystalline CTFs owing to the better separation of photogenerated electron–hole pairs and charge transfer.

**Figure 18:**
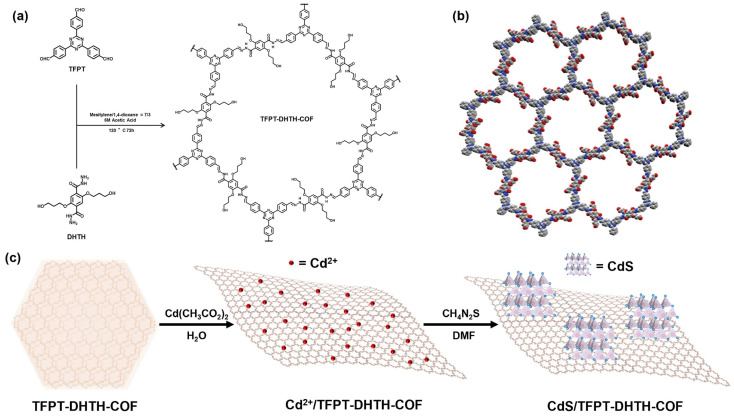
(**a**) Synthesis of TFPT-DHTH-COF. (**b**) Simulated crystal structures of eclipsed structure for TFPT-DHTH-COF. (**c**) Schematic synthetic process for CdS/TFPTDHTH-COF composite. (Reprinted with permission from [187], Copyright 2024, Elsevier).

For in situ growth of cadmium sulfide on a triazine based-COF, a direct Z-scheme heterojunction photocatalyst was developed [187]. The synthesis was accomplished by a polycondensation reaction using 2,5-bis-(3-hydroxypropoxy) terephthalohydrazide (DHTH) and 1,3,5-tris-(4-formyl-phenyl) triazine (TFPT). The resulting triazine-based covalent organic framework was designated as TFPT-DHTH-COF (Figure 18). Strong interfacial interactions with CdS occur at the nitrogen atoms in the TFPT-DHTH-COF structure. The mechanism of direct Z-scheme heterojunction photocatalyst hydrogen evolution was established by X-ray photoelectron spectroscopy and time-resolved photoluminescence measurements. It turned out that the Z-scheme charge transfer pathway is achieved as a result of strong interfacial interaction between TFPT-DHTH-COF and CdS. According to the measurements, the hydrogen evolution rate when using CdS/TFPT-DHTH-COF reaches 15.75 mmol h^−1^ g^−1^. For the TFPT-DHTH-COF sample, the hydrogen evolution rate is 75 times lower and is 0.21 mmol h^−1^ g^−1^. It is noteworthy that the hydrogen evolution rate of CdS is 3.4 times lower than that of CdS/TFPT-DHTH-COF.

**Figure 19:**
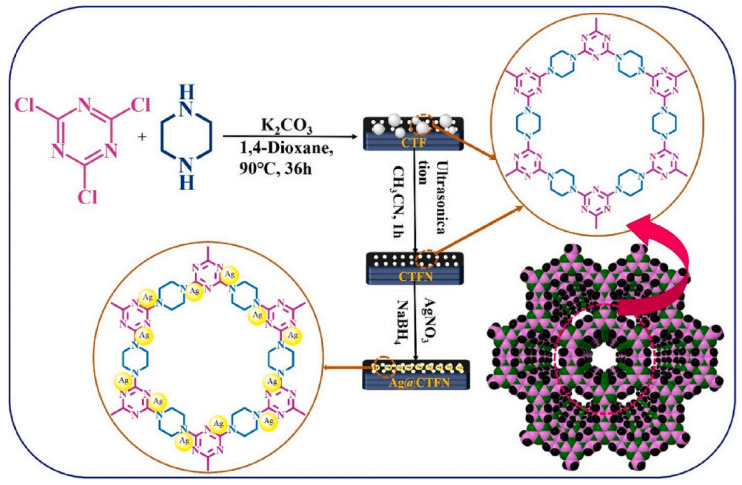
Synthesis of CTFN and Ag0@CTFN. (Reprinted with permission from [188], Copyright 2024, Elsevier).

To transform the nitrogen-rich but disordered structural state covalent triazine framework in its bulk form into an ordered structure in sheet form, ultrasonic treatment of the bulk covalent triazine framework in acetonitrile was carried out. Synthesis of CTFN nanosheets anchored with ultra-small-sized Ag nanoparticles was carried out using a one-pot strategy [188] (Figure 19). Due to the formation of uniform nitrogen sites, CTFN turned out to be a suitable framework for anchoring ultra-small sized Ag-NPs. They are important for the functioning of Ag-NPs, as the catalytic activity for the oxazolidinone synthesis from propargyl alcohol and amines by fixing CO_2_ is their stabilization from aggregation. Attachment of ultra-small sized Ag nanoparticles (4.5 nm in size) to the CTFN surface provides the necessary stability of these nanoparticles. The resulting catalyst can be reused for five cycles without losing activity and selectivity. Catalytic reactions were carried out at a temperature of about 28 °C and 1 atm CO_2_.

**Figure 20:**
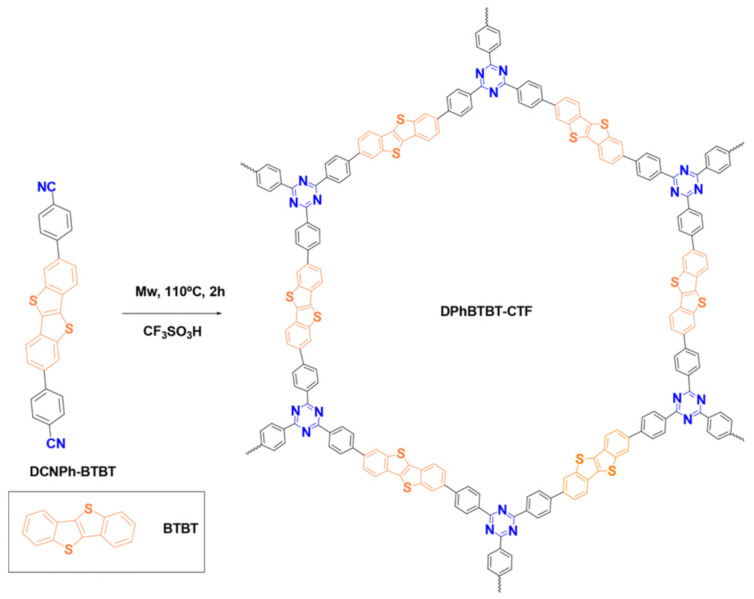
Synthesis and idealized structure of the DPhBTBT-conjugated covalent triazine framework. (Reprinted with permission from [189], 2024, The Royal Society of Chemistry).

Based on [1]benzothieno[3,2-b][1]benzothiophene (DPhBTBT-CTF), a covalent triazine framework was synthesized using an acid-catalyzed, microwave-activated cyclotrimerization reaction (Figure 20) [189]. Although DPhBTBT-CTF is amorphous, the coexistence of BTBT (electron donor) and triazine (electron acceptor) moieties has a synergistic effect producing an improved photocatalytic performance that does not occur with its molecular counterparts.

**Figure 21:**
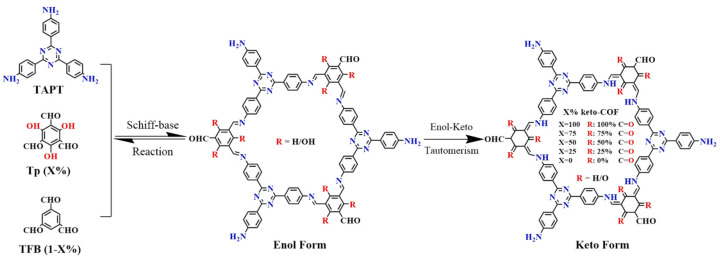
Synthesis and chemical structures of X% keto-CTF, where X represents the percentage ratio of trialdehyde phloroglucinol (Tp) monomer involved in the enol-to-keto tautomerization, X = 100, 75, 50, 25, 0. (Assuming that the positive reaction of the enol-to-keto tautomerization reaction defaults to 100%). (Reprinted with permission from [190], Copyright 2023, Elsevier).

To predict the performance of high-efficient visible-light photocatalysts using enol-to-keto tautomerization, X% keto-CTFs with an electron-rich ketone in the structure were synthesized (Figure 21) [190]. The structure-activity relationships for five CTFs differing in ketone moiety content estimated quantitatively using DFT. It was shown that increasing the ketone content quantitatively enhanced the photocatalytic performance of CTFs. The observed effect was explained by the regulation of the bandgap structure and the contribution of varied additionally formed active species.

A series of model CTFs with a controlled pore structure was prepared using ionothermal synthesis to study the effect of pore size and nitrogen content on the efficiency of using covalent triazine frameworks as supercapacitors [191]. It was found that the existence of mesopores is the reason for the increase in the contact area of the electrode material with the electrolyte and the subsequent boost of the charge transfer. The content of nitrogen as a heteroatom also has a noticeable effect on the improvement of the Faradaic pseudo-capacitance and conductivity for electrodes constructed using CTF. Thus, for BPY-CTF with a mesopore structure, optimal nitrogen content, and a specific surface area of 2278 m^2^ g^−1^, the specific capacity reaches 393.6 F g^−1^ at 0.5 A g^−1^.

**Figure 22:**
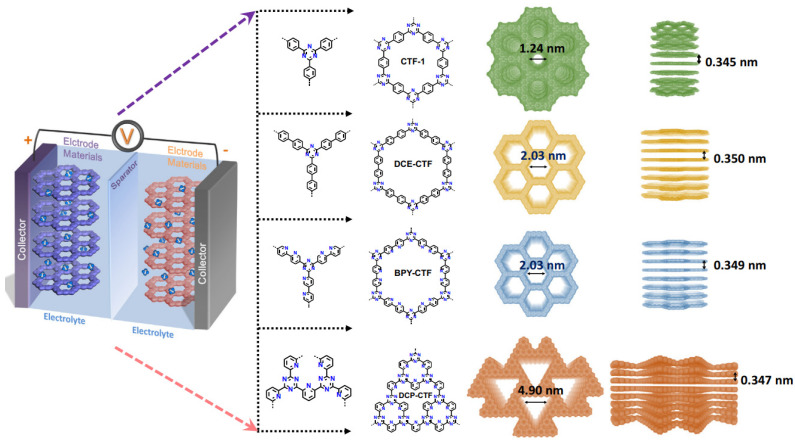
Schematic diagram of supercapacitor configuration together with diagram of design and synthesis of CTF-1, DCE-CTF, BPY-CTF, and DCP-CTF, including the top and side views of CTF-1, DCE-CTF, BPY-CTF, and DCP-CTF from left to right. (Reprinted with permission from [191], Copyright 2022, Elsevier).

The structure of conjugated CTFs differing in pore size and structure was constructed using ionothermal polymerization reactions of terephthalonitrile, [1,10-biphenyl]-4,40-dicarbonitrile, [2,20-bipyridine]-5, 50-dicarbonitrile, and pyridine-2,6-dicarbonitrile (Figure 22) [191]. The four building blocks chosen as monomers differ in molecular size, geometry, and number of nitrogen atoms.

### 2.5. Hypercrosslinked Polystyrenes

**Figure 23:**
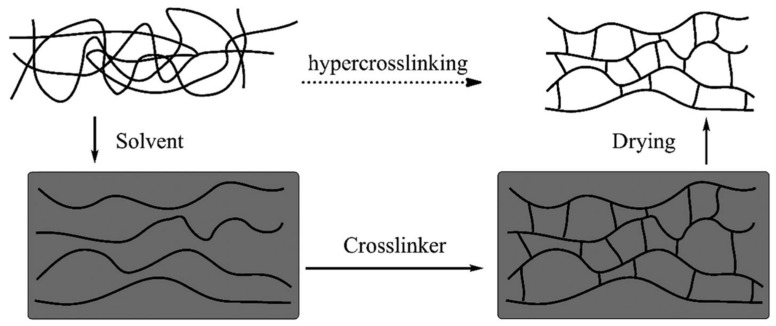
Schematic of post-crosslinking route to hypercrosslinked polystyrene network. (Reprinted with permission from [197], 2007, The Royal Society of Chemistry).

Among the micro- and mesoporous materials, an important place is currently occupied by hyper-crosslinked polystyrenes (HCPSt), developed in 1969 by V.A. Davankov and M.P. Tsyurupa [192,193,194,195,196]. The method was original and simple, since it was based on the crosslinking of the phenyl rings of polystyrene according to the well-known Friedel–Crafts reaction. The essence of the method lies in the crosslinking (hypercrosslinking) of the chains of linear polystyrene in a solvent that is thermodynamically good for polystyrene (Figure 23), which leads to the formation of a rigid, homogeneous, highly expanded spatial network. After removing the solvent, the space left by it (voids) is a porous structure [197]. Hypercrosslinked polystyrenes have acquired industrial significance. Thus, the company, Purolite International Ltd. (Llantrisant, Ynysmaerdy, Great Britain, UK) produces them under the brand name Hypersonal-Macronet.

The synthetic approach to obtaining HCPSt became the basis for the creation of other strategies for the synthesis of hypercrosslinked polymers of various types. To date, many hypercrosslinked polystyrene have been synthesized that have unique physical properties, such as good permeability, osmotic stability, chemical and hydrolytic resistance, high sorption capacity with respect to many organic compounds, and are distinguished by a developed porous structure with an extremely high specific internal surface area (~600–2000 m^2^/g). The greatest number of studies are devoted to the creation of microporous sorbents. Such materials are mainly focused on their use in processes associated with the sorption of small molecules with an aromatic structure. Fewer studies are devoted to the production of (HCPSt) with differentiated porosity, and very few studies are associated with the production of mesoporous and biporous sorbents for the sorption of sufficiently bulky molecules, such as antibiotics or low-molecular polyphenols or flavonoids.

**Figure 24:**
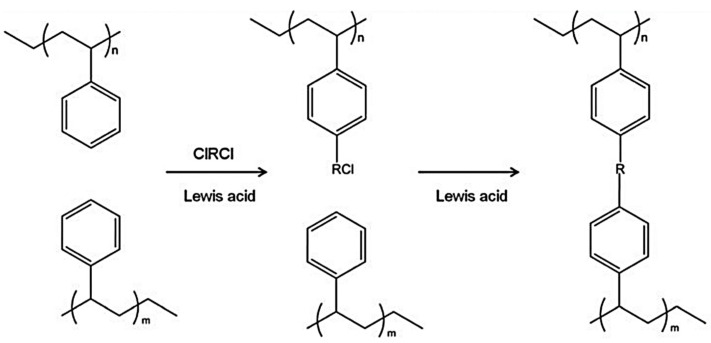
Basic synthetic route of hyper crosslinked polymers: polymers with aromatic side chains are linked using a high concentration of methylene dihalides in the presence of a Lewis acid catalyst, to create a high concentration of cross-links. (Reprinted with permission from [198], 2017, The Royal Society of Chemistry).

In [198,199,200], microporous hypercrosslinked organic polymer networks were developed as hydrogen storage. Despite the diversity of existing strategies for the synthesis of HCPSts, which have been strongly developed in recent decades, their basis is the Friedel–Crafts reaction. It is an alkylation of aromatic compounds with halogen alkyls, α-halogen-activated ethers, or acetals (electrophilic substitution in the aromatic ring), occurring in the presence of Lewis acids (Figure 24) [198].

In the synthesis of HCPSt by the Friedel–Crafts reaction, strong Lewis acids (AlCl_3_, FeCl_3_, SnCl_4_, ZnCl_2_, BCl_3_) are used as catalysts. In [201,202], it was established that for this series of Lewis acids, FeCl_3_ exhibits the greatest activity.

An effective crosslinking agent for obtaining HCPSt is monochlorodimethyl ether [203]. Tetrachloromethane [204,205,206], dimethoxymethane [207,208,209], cyanuric chloride [210], and trimethyl orthoformate [211] are also known as crosslinking agents. During the hypercrosslinking process, bridging groups are formed that hold the macrochains at the distance necessary for the formation of micropores. At the same time, the mobility of such bridges is preserved.

The choice of a solvent, which should have good solvating capacity, and the possibility of the Friedel–Crafts reaction occurring in its medium, is an important condition for obtaining a microporous structure in HCPSt. The most frequently used solvent is 1,2-dichloroethane [193].

**Figure 25:**
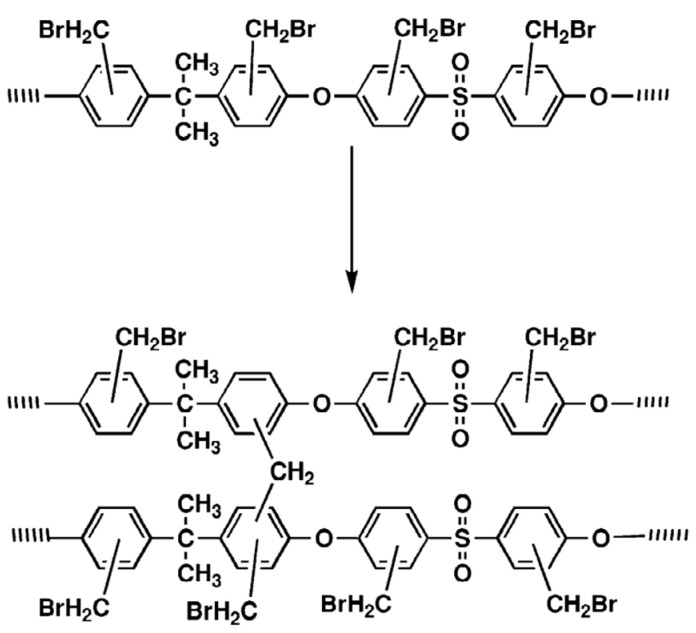
Crosslinking of polysulfone. (Reprinted with permission from [212], Copyright 2024, Elsevier).

The post-crosslinking strategy has been used to prepare other hypercrosslinked polymers using polysulfone, polyarylate, polyaniline, and polypyrrole [212,213,214]. Thus, hypercrosslinked polysulfones were obtained by the Friedel–Crafts reaction by crosslinking bromomethylated polysulfone [212] (Figure 25).

## 3. Porous Coordination Polymers

One of the promising methods for creating microporous polymers is the synthesis of metal–organic frameworks (MOFs). In metal–organic frameworks, metal ions are the nodal element of the framework. As a result of the rapid development of modular design, MOFs held together by coordination bonds can be linked in a predictable manner. This then allows manipulation of the functional units and spatial structures of the framework, thereby allowing control over the internal surface engineering and functionalization of the MOFs [215,216]. MOFs are excellent candidates for high performance gas storage and separation applications owing to their tunable pore size and modifiable pore environment [217,218,219,220,221,222,223,224,225,226].

In the case of porous coordination polymers (PCPs), metal ions act as linkers between organic rigid blocks. PCPs represent one of the most attractive classes of porous materials at present [227,228,229,230].

**Figure 26:**
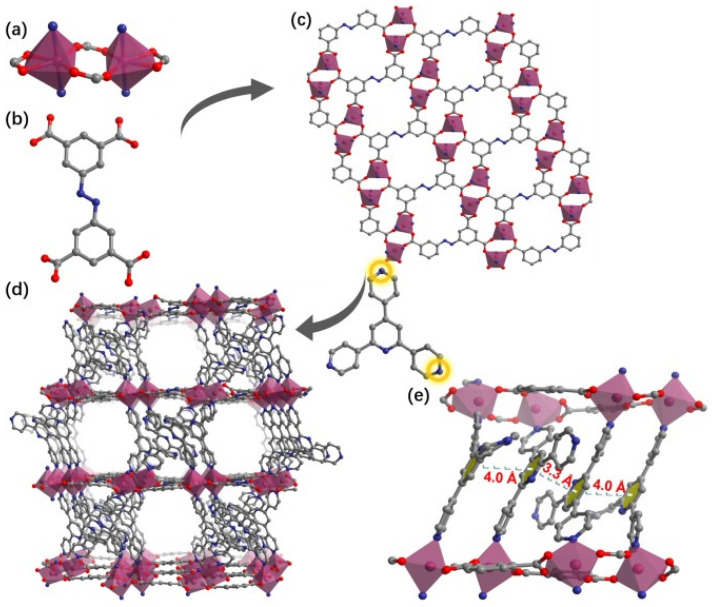
Inorganic [Co_2_(COO)_3_N_2_] cluster; (**b**) ABTC^4−^ linker; (**c**) View of the 2D layer constructed from ABTC^4−^ and Co (II) centers; (**d**,**e**) 3D pillar-layer framework of YTU-30 with narrow channels. (ABTC^4−^ is the anion of the 3,3′,5,5′-azobenzene tetracarboxylic acid). (Reprinted with permission from [231], Copyright 2024, Elsevier).

A mixed ligand strategy and the introduction of dense aryl rings as bridging ligands was adopted for the cobalt-based synthesis of a novel microporous “pillar-layer” MOF (YTU-30) for the SF_6_ and N_2_ separation from their gas mixtures (Figure 26) [231]. A distinctive feature of the YTU-30 structure is the existence of multiple π–π stacking interactions between dense aryl rings. Using the ideal adsorbed solution theory, measurements of dynamic breakthrough curves and separation potential, the effective capture of SF_6_ from binary gas mixtures of SF_6_ and N_2_ as significant chemical stability in an aqueous medium at pH 3~11 were confirmed. The high ability to separate SF_6_ from gas mixtures according to Grand Canonical Monte Carlo simulations is primarily due to the dense aryl rings on the pore inner wall. The aryl structure of the pore cavity promotes the adsorption of SF_6_ molecules through S-F-π and C-H⋅⋅⋅F interactions.

**Figure 27:**
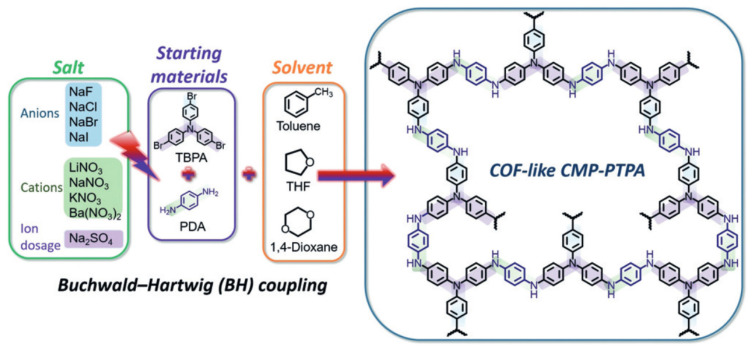
Synthetic route for the formation of salt-tunable PTPA networks. (Reprinted with permission from [232], 2019, Wiley).

Poly(triphenylamine) (PTPA) was synthesized by Buchwald–Hartwig cross-coupling reactions (Figure 27) [232]. Alkali metal salts were used to tune N-containing CMPs. PTPA was synthesized without using inorganic salts characterized by a broad distribution of micropore, mesopore, and macropore sizes. However, by changing the sizes of anions and cations and their variations, fine tuning of the surface area of PTPA becomes possible. With an increase in the ionic radius of anions and cations, a corresponding decrease in the surface area occurs, and the pore size distribution narrows exclusively to the microporous range. The surface area undergoes a significant increase from 58 m^2^ g^−1^ to 1152 m^2^ g^−1^ and becomes similar to COF and MOF. Adjustment and optimization of Hansen solubility parameters under the influence of salt explains the observed effect on the physical properties of the polymer and is named the Beijing–Xi’an Jiaotong method.

**Figure 28:**
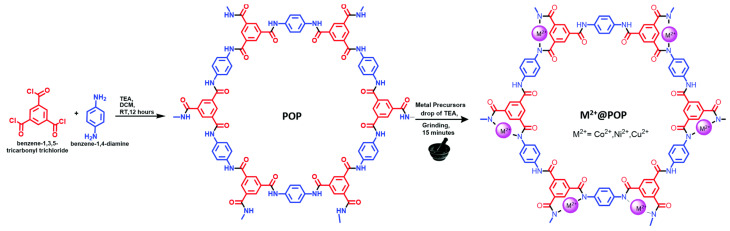
Schematic illustration for synthesis of M^2+^@POP. (Reprinted with permission from [233], 2022, The Royal Society of Chemistry).

The incorporation of M^2+^ into the structure of porous organic polymers was found to be an important factor preventing leaching and agglomeration [233]. Synthesis of POPs coordinated by transition metal ions was accomplished by a mechanochemical method (Figure 28). Prepared Cu^2+^@POP, Ni^2+^@POP, and Co^2+^@POP in the presence of H_2_O_2_ were found to be highly active in the tetramethylbenzidine (TMB) oxidation reaction, during which uncolored TMB was converted into blue ox-TMB as a result of one electron transfer. The peroxidase-like activity of Cu^2+^@POP, which prevented TMB oxidation in the presence of ascorbic acid (AA) and dopamine (DA), was found to be important. Cu^2+^@POP exhibited the highest catalytic activity among the synthesized material coordinated by transition metal ion POPs and can be used as biosensors. Colorimetric detection of ascorbic acid and dopamine achieved low detection limits of 1.4 mM and 1 mM for AA and DA.

## 4. Polymers with Intrinsic Porosity

A typical approach to creating polymers with intrinsic porosity (PIM) is to combine two or more rigid polymer fragments into three-dimensional spatial structures. If hypercrosslinked polymers are structurally similar to a rigid sponge, then polymers with intrinsic porosity are like a basket filled with broken glass fragments—large and flat fragments cannot be packed without leaving voids. Polymers with intrinsic microporosity are a rare example of porosity in a one-dimensional polymer, achieved due to inefficient packing of a twisted polymer chain [234,235,236,237,238,239,240].

**Figure 29:**
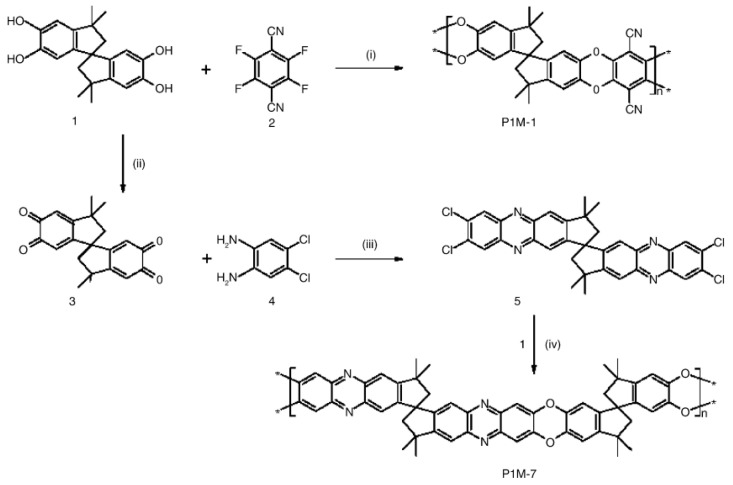
Preparation and structures of polymers PIM-1 and PIM-7. Reagents and conditions: (i) K_2_CO_3_, DMF, 65 °C; (ii) Conc. HNO_3_, HOAc; (iii) HOAc, reflux; (iv) 18-crown-6, K_2_CO_3_, DMF, 150 °C. (Reprinted with permission from [236], Copyright 2005, Elsevier).

One of the first representatives of polymers with intrinsic microporosity is PIM-1. The main studies of PIM-1 and its structural analogs were aimed at their use as membrane materials [236,237,238,239,240]. PIM-1 was synthesized by a relatively simple polycondensation method (Figure 29) [236].

**Figure 30:**
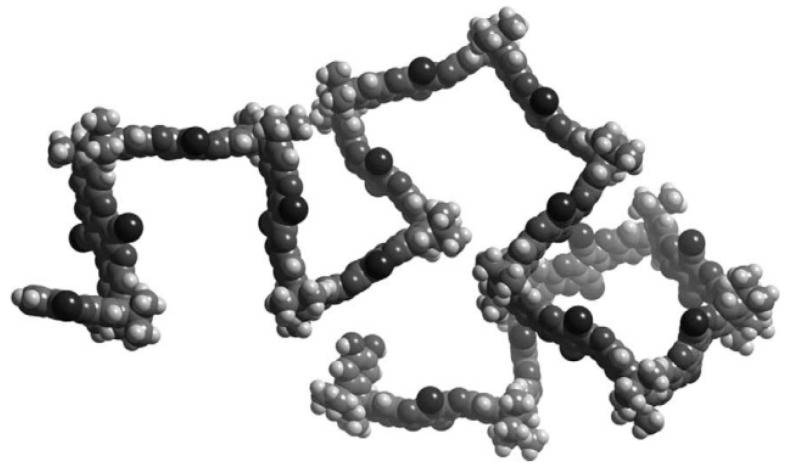
Molecular model of a fragment of PIM-1 showing rigid, contorted structure. (Reprinted with permission from [236], Copyright 2005, Elsevier).

The backbone of PIM-1 and PIM-7, as shown in Figure 29, lacks single bonds to provide rotation. However, one tetrahedral carbon atom shared by two rings at intervals along the backbone creates a spiro-center and thus introduces a sharp bend into the chain (Figure 30). These polymers are in an amorphous (glassy) state up to their thermal degradation temperature (>350 °C).

PIM-1 has higher solubility coefficients than any of the studied polymers and therefore high gas permeability coefficients. An important characteristic of PIM-1 is a good combination of permeability and permeability selectivity, therefore the data points on several Robson diagrams are located above the upper limits. PIM-1 has a large free volume [239] and a high specific internal surface area (about 700–900 m^2^/g). An interesting feature of PIM-1 and its analogs is an almost tenfold increase in gas permeability coefficients after swelling of films in methanol and ethanol [239].

**Figure 31:**
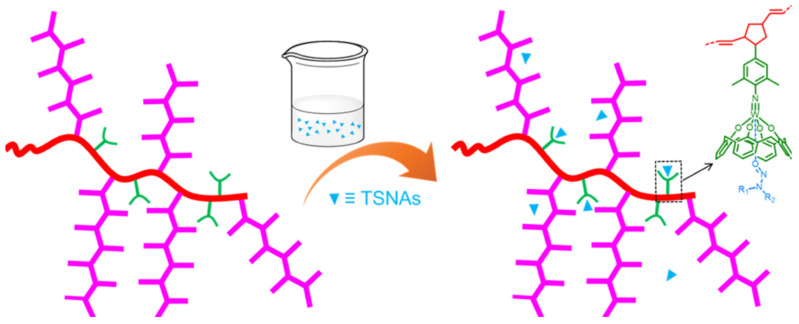
Porous polymers containing metallocalix[4]arene for the extraction of tobacco-specific nitrosamines (TSNAs). (Reprinted with permission from [241], 2022, American Chemical Society).

Another method for obtaining polymers with intrinsic porosity is the synthesis of polymers based on molecules capable of forming host–guest complexes, such as cyclodextrins or calixarenes [241] (Figure 31).

**Figure 32:**
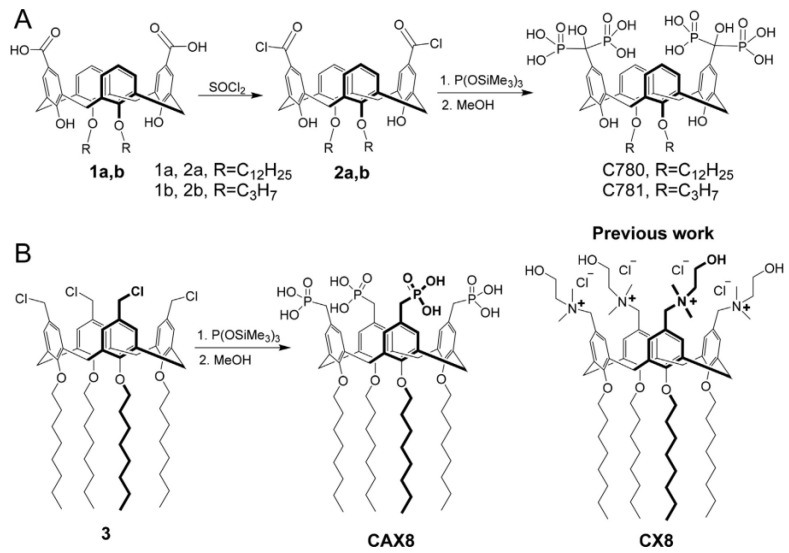
Schemes of synthesis of C780, C781 (**A**) and CAX8 (**B**) and structure of CX8. (Reprinted with permission from [242], Copyright 2022, Elsevier).

Amphiphilic calixarenes have been synthesized, in the structure of which there are four phosphonate groups linked to long alkyl chains (four or two) (Figure 32). These calixarenes, existing in the micromolar concentration range, form micelles, while amphiphilic calixarenes with two short alkyl chains were found to be incapable of forming micelles [242].

The anionic amphiphilic calixarenes synthesized in [242] used as nanocarriers for peptides and their interaction with cells was studied. Amphiphilic anionic calixarenes turned out to be interesting objects for self-assembly and forming micelles capable of complex cationic peptides into small nanoparticles. Anionic amphiphilic calixarenes showed low cytotoxicity and are promising for the delivery of cationic peptides into cells.

Ion-selective electrodes (ISEs) are widely used in pharmaceuticals, biofluids, and environmental analysis. Ionophore is a key component in membrane production. It is responsible for the desired selectivity in ISEs. The host–guest chemistry represented by the ionophore–analyte interaction explains the best choice of ionophore. This leads to low detection limits and increased sensitivity of the fabricated sensors.

**Figure 33:**
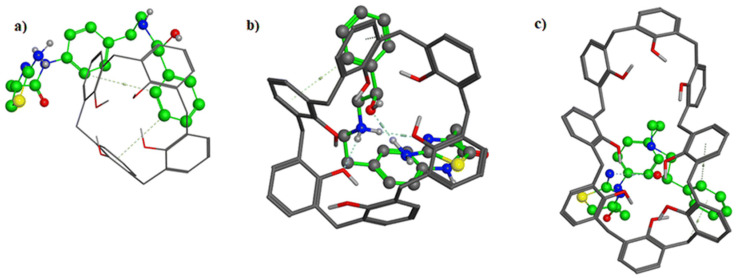
Three-dimensional docking of mirabegron in (**a**) calix[4]arene, (**b**) calix[6]arene, and (**c**) calix[8]arene. (Reprinted with permission from [243]. Copyright 2023, The Royal Society of Chemistry).

A portable solid-contact potentiometric electrode was designed for the sensitive determination of mirabegron (MIR) in human plasma and pharmaceutical formulation [243]. A two-step optimization protocol was investigated for the fabrication of an ion on sensing polymeric membrane. Molecular docking was used for optimum ionophore selection. Calix[6]arene (Figure 33) showed the highest affinity towards MIR with a better docking score (−4.35) and potential energy (−65.23) compared to other calixarene derivatives.

**Figure 34:**
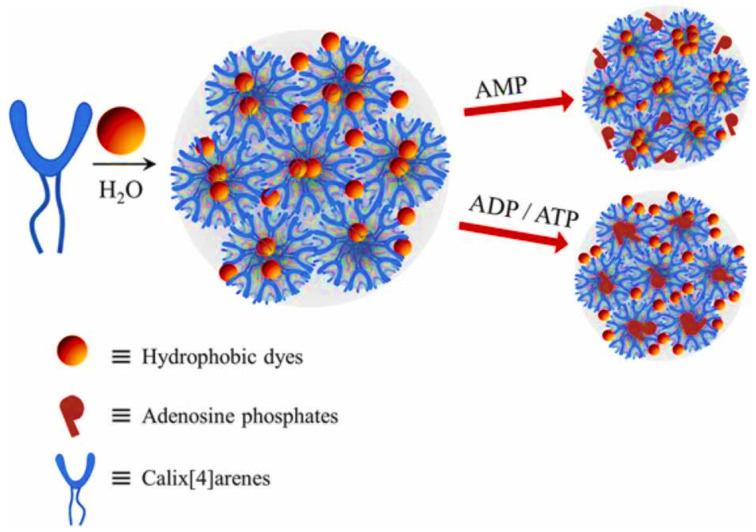
Amphiphilic N-oxyethylimidazolium calixarenes. (Reprinted with permission from [244]. Copyright 2022, Elsevier).

The conical stereoisomeric N-hydroxyethylimidazolium-modified amphiphilic calixarenes were synthesized by stepwise modification of calix[4]arene (Figure 34) [244]. The critical micelle concentration of the obtained calixarenes was determined. The stable formation of the hydrophobic core of the macrocycles ensures the use of pyrene. Application of these amphiphiles is aimed at creating nonviral vectors and delivery vehicles for hydrophobic biologically active vehicles.

## 5. Block Copolymers in the Creation of Microporous and Mesoporous Polymers

**Figure 35:**
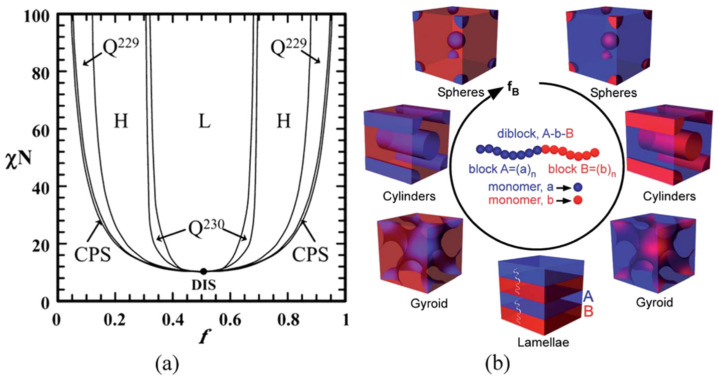
(**a**) Phase diagram of diblock copolymer predicted by SCMF theory (reprinted with permission from [245], Copyright 2006, American Chemical Society); (**b**) Various microdomain organization patterns of a linear AB diblock copolymers (reprinted with permission from [246], Copyright 2014, The Royal Society of Chemistry); f: volume fraction of one block; χ: Flory–Huggins interaction parameter; N: degree of polymerization; L: lamellae; H: hexagonally packed cylinders; Q230: double gyroid phase; Q229: body centered spheres; CPS: closed-packed spheres; and DIS: disordered.

The production of porous structures can also be based on the processes of self-organization of block copolymers (BCs). Although the porous structure of microporous polymers is usually disordered, new ordered ones with a crystalline framework structure have recently been rapidly developing. The creation of polymers with tunable sizes on the nanometer scale can also be based on the self-organization ability of BC consisting of two or more covalently bonded polymer blocks. The thermodynamic incompatibility of BC blocks is due to the difference in their chemical nature and the low entropy of mixing per unit volume, which changes inversely proportional to the increase in the molecular weight of the blocks. As a result, it becomes possible to create complex nanostructures that, depending on the relative volume fraction of one block with respect to another, can exhibit a wide range of morphologies. The result is spontaneously organized periodic nanostructures including sphere, cylinder, gyroid, and lamellae with tunable periodicity, typically in the range of 5–100 nm (Figure 35) [245].

**Figure 36:**
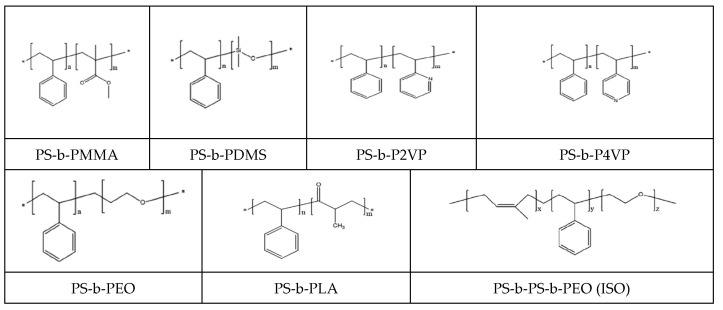
Representative block copolymers for nanopatterning. (Reprinted with permission from [247], Copyright 2022, American Chemical Society).

BCs are widely used in various technological fields mainly as thermoplastic elastomers, primarily butadiene-styrene, which occupy the largest share of the commercial market for their use. With the advent of nanotechnology, the self-assembly of BCs have been studied in solutions, solids, and thin films. Below are the structures of some BCs, where polystyrene blocks are used as rigid-chain components [247] (Figure 36).

**Figure 37:**
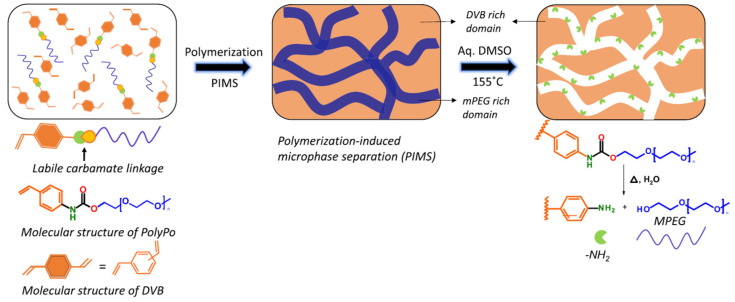
Schematic representation of the process for generation of internally functionalized mesoporous monoliths by copolymerization of a thermally labile polymerizable porogen (PolyPo) and divinyl benzene (DVB). As the polymerization proceeds, the MPEG segments undergo microphase separation via a process similar to polymerization-induced microphase separation (PIMS); solvothermal treatment with alkaline aqueous-DMSO (10 wt % NaOH-water in DMSO) at 155 °C leads to the cleavage of the urethane linkage and in situ hydrolysis of the isocyanate to yield amine functional groups that line the internal walls of the pores. MPEG-OH formed as a result is removed by washing. (Reprinted with permission from [248], Copyright 2021, American Chemical Society).

The chemical structure and mobility of the monomer units of polystyrene blocks make it difficult to form voids during microphase separation of BCs. From the point of view of obtaining microporous polymers, block copolymers have proven to be convenient objects for creating nanotemplates—universal tools for nanoscale production for semiconductor devices—and other applications due to their ability to organize clearly defined periodic nanostructures with a critical size of 5–100 nm. Figure 37 shows an example of such technologies used to obtain microporous polymers [248].

Although the most promising areas for nanopatterning using BCs are semiconductor devices, the versatility of BCs has attracted a wide range of applications in this capacity. In particular, the initially low cost and simple technology for creating BC-based nanopatterns has made it possible to form dense nanoscale features over a large area, which is in stark contrast to the complex processing steps of a typical photolithographic process, including deep ultraviolet lithography [247].

### Block Copolymers Obtained Using Aromatic Isocyanates

**Figure 38:**
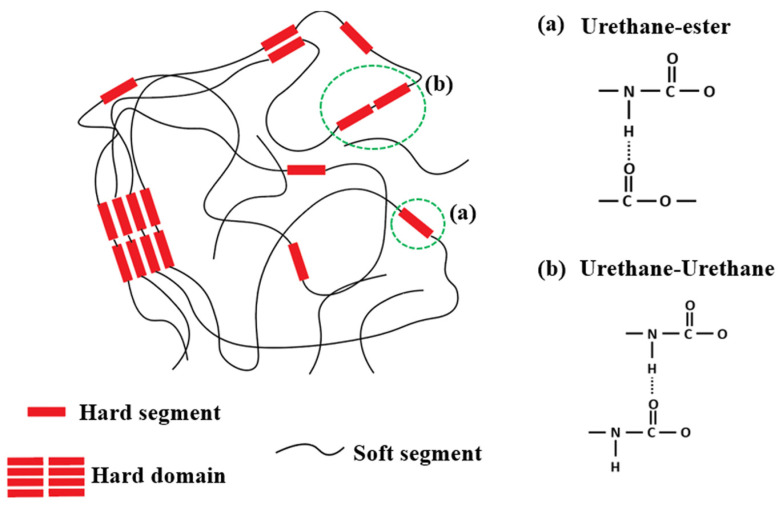
Schematic representation of hydrogen bonding in thermoplastic polyurethane elastomer. (Reprinted with permission from [258], Copyright 2023, Elsevier).

The ability to undergo microphase separation is also due to the chemical structure of segmented polyurethanes (SPU). Segmented polyurethanes are alternating block copolymers consisting of flexible soft segments and rigid segments (Figure 38) [249,250,251,252,253,254,255,256,257,258,259,260].

As a rule, a wide range of oligoetherdiols are used as a flexible-chain component for their synthesis. Symmetrical aromatic diisocyanates—4,4-methylene diphenyl diisocyanate and 1,5-naphthylene diisocyanate and a short-chain diol (chain extender)—are used to create hard blocks. Changes in the compositions of soft and hard segments impart a wide range of physical properties to polyurethanes. In this regard, the relationship between the PU structure and such properties as vapor permeability, which could be a consequence of the occurrence of micropores in segmented PU, is of interest. However, intermolecular voids arising due to the non-ideal loose packing of SPU are much smaller than micropores and are invisible even to a scanning electron microscope due to the resolution limit of the microscope [250].

Dense polymer membranes are non-porous, but there is thermally excited motion of chain segments that creates temporary gaps (free volume) in the polymer matrix [261]. The free volume creates fluctuating holes of a certain shape and size that control the transport of molecules through the dense polymer membrane [249,262,263]. As a result of thermally excited segmental motion, diffusion of penetrants through non-porous dense polymer membranes becomes possible, and the diffusion characteristics will be determined by the free space. The concept of the free volume unoccupied by polymer chains at the microscopic structural level explains the molecular transport through such membrane materials [264]. Positron annihilation lifetime spectroscopy is one of the main methods for studying subnanometer holes and allows determining not only their volume but their size distribution [265]. In the case of membranes based on segmented polyurethanes, the diffusion characteristics of water vapor was tuned by controlling the free volume. Thus, the vapor permeability and gas permeability of membranes obtained using SPU will depend on their chemical composition and hydrophilicity [266,267,268,269,270].

The possibilities of creating new macromolecular architectures by influencing the reactivity of isocyanate groups (NCO) of unsymmetrical 2,4-toluene diisocyanate (TDI) are described in our works [271,272,273,274].

**Figure 39:**
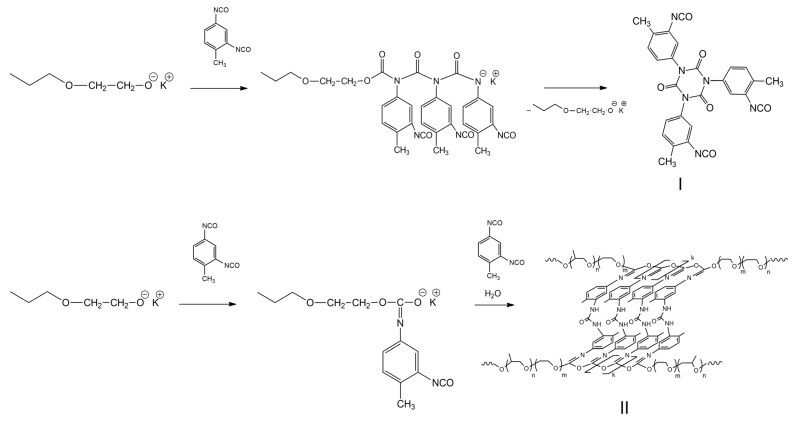
Scheme of PPEG and TDI interaction. (Reprinted with permission from [278], Copyright 2023, MDPI).

Isocyanate groups can be involved in migration and homopolymerization reactions, and also react with each other with subsequent formation of dimers and trimers. The reaction development pathways and formation of final products are determined by the isocyanate activation method. The use of partially terminated potassium-alcoholate groups of block copolymers of propylene and ethylene oxides (PPEG, MM = 4200) as anionic macroinitiators has proven promising in this direction. The significant influence of reaction conditions on the conformational behavior of the latter and, accordingly, on the reactivity of terminal potassium-alcoholate groups has become a prerequisite for controlling the structure and properties of polymers obtained by their activation action on aromatic isocyanates. According to the literature, the opening of NCO groups at the N=C bond leads to the formation of polyisocyanates of amide nature, which, in the absence of active center stabilizers, undergo cyclization with subsequent formation of isocyanurates and polyisocyanurates (Figure 39, structure I). The fact of an unusual opening of isocyanate groups at the carbonyl component, leading to the formation of polyisocyanates of acetal nature (O-polyisocyanates), discovered by a number of researchers, is interesting, but practically unstudied. Thus, polyisocyanates having an exclusively polyacetal chain structure have not yet been obtained and characterized in detail. The flat spatial configuration of O-polyisocyanate segments in combination with the amphiphilicity of PPEG seems to be an important element in the design of the supramolecular structure of polymers with their participation (Figure 39, structure II) [275,276,277,278].

The conducted studies allowed us to establish that water in combination with triethylamine is the cause of the involvement of NCO groups of the *ortho*-position of neighboring O-polyisocyanate blocks in the formation of urea, causing their segregation. As a result, the self-assembly of O-polyisocyanate blocks is carried out not as a result of intermolecular interactions of a physical nature, but as a consequence of the binding of neighboring O-polyisocyanate blocks by means of urea groups (Figure 39 structure II).

The possibility of forming either O-polyisocyanate units or polyisocyanurates in the reaction system under study is determined by such reaction conditions as the synthesis temperature, the nature and content of the solvent, the presence of acidic cocatalysts, the molecular weight of PPEG, the molar fraction of its polyethylene oxide segments, and the molar ratio [PPEG]/[TDI].

Predominant formation of O-polyisocyanates and, accordingly, block copolymers with their participation is the reason for a noticeable decrease in the specific volume electrical resistance of polymers obtained on the basis of PPEG and TDI [279].

The sorption capacity of POI turned out to be much higher compared to PEI. Nevertheless, the values of water sorption of PEI also suggest the possibility of forming pores of transition sizes here. As a result, by changing the conditions of interaction of PPEG and TDI, it turned out to be possible to obtain optically transparent mesoporous polymers that differ in the chemical structure of the surface of the voids. The processes of laser radiation generation and photochemical aging of xanthene and merocyanine dyes in PEI and nonporous polymers were studied. It was found that organic luminophores immobilized on PEI film samples exhibit the ability to generate laser radiation, and high radiation stability in comparison with polymethyl methacrylate was used for these purposes [280,281,282,283].

The formation of a polymer matrix with a predominant content of O-polyisocyanate blocks (POI) is the reason for their self-organization with the formation of a cellular supramolecular structure [271]:


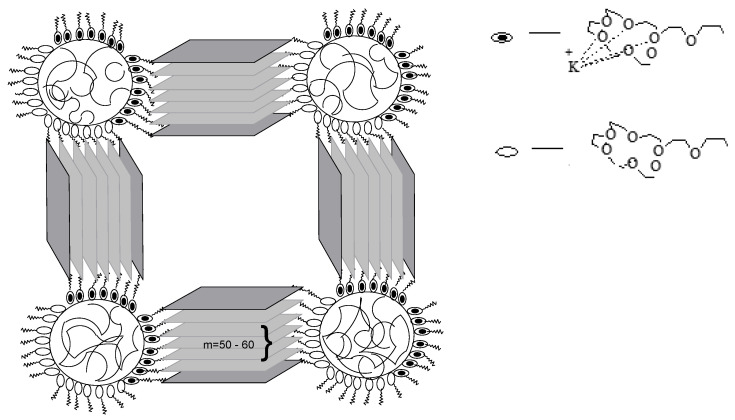


In the case of the predominant formation of polyisocyanurates, the supramolecular structure of the corresponding PEI is of the core-shell type [271]:


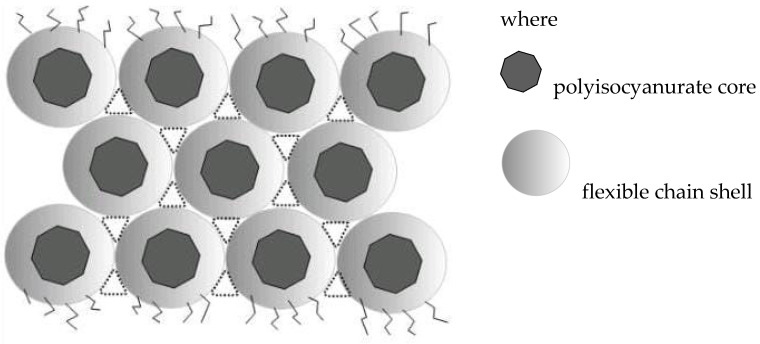


**Figure 40:**
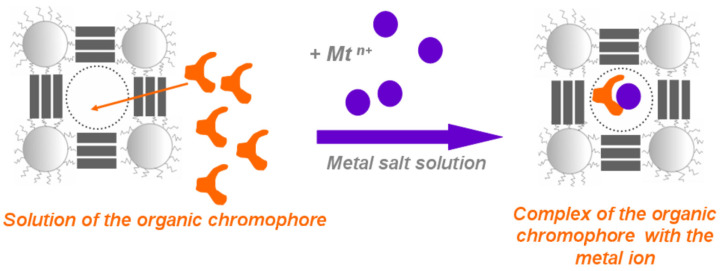
Scheme of the reaction of complexation of an organic reagent with metal cations. (Reprinted with permission from [283], Copyright 2014, IntechOpen Limited).

The efficiency of PEI sorption of organic reagents was studied [275]. It was found that the highest values of the concentration coefficient correspond to mesoporous PEI. Using model solutions containing Cu(II) and Ca(II) as an example, it was shown that complexation of metal ions with immobilized organic reagents is possible in the void cavity (Figure 40). The results of quantitative analysis obtained using a polymer substrate are not inferior in terms of the minimum detectable concentration to measurements carried out in solutions of organic reagents and are 20 μg/mL for calcium ions and 2 μg/mL for copper ions [276,277,278,282,283].

Studies have been conducted to determine the effect of the content of polyethylene oxide blocks (POE) in PPEG on the formation of the supramolecular structure of PPEG and their properties [279]. With a decrease in the content of POE blocks in the PPEG, the flexible-chain component is drawn into its internal space. In this case, microphase separation occurs not only on the flexible-chain and rigid-chain components, but also results in the release of propylene oxide and POE blocks into their own microphase. An increase in the content of peripheral POE blocks in the PPEG leads to a change in the morphology of the POI surface and a decrease in their sorption activity.

For polymers obtained under the selected reaction conditions, gas transport characteristics were studied [280]. When obtaining polymer membranes, the main task is to create a material that could combine high selectivity with high permeability. The gas transport properties of POI obtained with different POE content in the initial PPEG were studied. He, N_2_, NH_3_, CH_4_, H_2_S, and CO_2_ served as gaseous substances. It was found that the permeability through POI for polar molecules of hydrogen sulfide and ammonia significantly exceeds the permeability obtained for non-polar molecules of He, N_2_, and CH_4_. Relatively high permeability values are also observed for carbon dioxide. At the same time, the content of POE blocks has a slight effect on the permeability values through POI for all the gases studied. During the tests, high selectivity values were achieved for gas mixtures containing polar and non-polar molecules. It was shown that for the gases used in the studies, the diffusion coefficient increases with an increase in the proportion of POE blocks in the PPEG. The obtained regularities are consistent with the assumption that the pore cavity of POI obtained with a low content of POE blocks is filled with a POE component. As a result, the volume of space inside the pores decreases. In addition, an obstacle appears for gases due to the imperfection of the alignment of the channels connecting the voids.

## 6. Conclusions

Synthesis of organic polymers with micro- and mesoporous structure is a current direction of polymer chemistry. The main areas of application of porous polymers include materials for gas storage and separation, encapsulating agents for controlled drug release, as carriers for catalysts and sensors, templates for creating nanostructured carbon materials, carriers for biomolecular immobilization and cellular frameworks, materials with low dielectric constant, filtration/separation membranes, proton exchange membranes, templates for replicating structures, electrode materials for energy storage, among many other applications. Sol–gel technologies, track etching, and template synthesis are used for their production, including in micelles of surfactants and microemulsions and sublimation drying. However, all these methods are technologically multi-stage and require the use of consumable components, which leads to low efficiency of using the initial materials, and the final product does not have micropores. These disadvantages are absent in the methods of synthesizing micro- and mesoporous polymers based on macromolecular architecture.

In this review, we discuss many examples of recent progress in the use of macromolecular architecture in the synthesis of micro- and mesoporous polymers with extremely high surface areas and hierarchical porous polymers, as well as the synthesis of porous polymer frameworks with individual functional capabilities, the required chemical structure, and pore surface sizes based on the unique possibilities of developing the architecture of the polymer matrix.

## Data Availability

The original contributions presented in the study are included in the article, further inquiries can be directed to the corresponding author.
